# Gray and White Matter Networks Predict Mindfulness and Mind Wandering Traits: A Data Fusion Machine Learning Approach

**DOI:** 10.3390/brainsci15090953

**Published:** 2025-09-01

**Authors:** Minah Chang, Sara Sorella, Cristiano Crescentini, Alessandro Grecucci

**Affiliations:** 1 Department of Psychology and Cognitive Sciences, University of Trento, 38068 Rovereto, Italy; minah.chang@unitn.it; 2 Department of Languages and Literatures, Communication, Education and Society, University of Udine, 33100 Udine, Italy; sara.sorella@uniud.it (S.S.); cristiano.crescentini@uniud.it (C.C.); 3 Centre for Medical Sciences, University of Trento, 38122 Trento, Italy

**Keywords:** mindfulness, spontaneous/deliberate mind wandering, acting with awareness, structural MRI, data fusion networks, parallel independent component analysis

## Abstract

Background: Mindfulness and mind wandering are cognitive traits central to attentional control and psychological well-being, yet their neural underpinnings are yet to be elucidated. This study aimed to identify structural brain networks comprising gray matter (GM) and white matter (WM) that predict individual differences in mindfulness and distinct mind wandering tendencies (deliberate and spontaneous). Methods: Using structural MRI data and self-report measures from 76 participants, we applied an unsupervised data-fusion machine learning technique (parallel independent component analysis) to identify GM and WM networks associated with mindfulness and mind wandering traits. Results: Our analysis revealed several distinct brain networks linked to these cognitive constructs. Specifically, one GM network involving subcortical regions, including the caudate and thalamus, positively predicted mindfulness and deliberate mind wandering, while negatively influencing spontaneous mind wandering through the mediating role of the mindfulness facet “acting with awareness.” In addition, two separate WM networks, predominantly involving frontoparietal and temporal regions, were directly associated with reduced spontaneous mind wandering. Conclusions: These findings advance our current knowledge by demonstrating that specific GM and WM structures are involved in mindfulness and different forms of mind wandering. Our results also show that the “acting with awareness” facet has a mediating effect on spontaneous mind wandering, which provides supporting evidence for attentional and executive control models. These new insights into the neuroanatomical correlates of mindfulness and mind wandering have implications for ongoing research in the growing topic of mindfulness and mind wandering, mindfulness-based interventions, and other clinical applications.

## 1. Introduction

### 1.1. Background

Our attention is engaged in the present moment by immediate environmental stimuli that we perceive through the senses, and also by interoceptive cues such as hunger, pain, sleepiness, and sexual desire. On the other hand, our neurocognitive processes are not restricted to such immediacy [1,2]. Rather, the mind wandering away from conspicuous sensations and perceptions is considered the normal psychological baseline [3] during 25 to 50 percent of our waking hours [4,5], especially when resting or sleeping, working, using a home computer, and commuting or traveling [5]. The self-generated thoughts of mind wandering that arise intrinsically within an individual can be either deliberate (intentional) or spontaneous (unintentional), and they can also be characterized as adaptive or maladaptive [6,7].

Since the executive components of attention shift away when the mind wanders from the current environment, mind wandering shares many similarities with traditional executive models of attention [2,8]. According to control failure hypothesis, the mind wandering away from task-related thoughts could be a result of failure of executive control to keep on the current task [9,10]. In support of this theory, the self-generation and disengagement of mind wandering are influenced by individual differences in executive control [4,9].

Mindfulness is largely composed of two different components: self-regulation of attention, and adopting a particular orientation toward one’s experience in the present moment as characterized by curiosity, openness, and acceptance [11,12,13]. Therefore, mindfulness is also linked to attention and executive control as is mind wandering, though exerting its influence in the opposite direction. Structural evidence suggests that mindfulness training results in plasticity of brain mechanisms involved in emotional regulation through cognitive top-down emotion regulation (i.e., through the control of prefrontal cortex (PFC) to inhibit the influence of limbic areas, such as amygdala) in short-term training, but with perceptual bottom-up emotion regulation with long-term training [14,15,16]. The top-down emotion regulation in short-term training might be characterized as more deliberate cognitive control at a conscious level, and the bottom-up emotion regulation in long-term training might be characterized as more automatic control occurring below the level of conscious control. The latter would translate into a more stable trait that persists over time. Future studies may reveal the parallelism between top-down processes of mindful emotion regulation and deliberate mind wandering on the one hand, and bottom-up mindful emotion regulation and spontaneous mind wandering on the other.

Studies that examined the effects of short-term mindfulness training found that mind wandering tendencies can be significantly reduced even in as short as a two-week period [17,18]. Structural evidence shows that just 2 to 4 weeks of mindfulness training result in increased myelin and other axonal changes in white matters surrounding the anterior cingulate cortex (ACC) and posterior cingulate cortex (PCC) [19,20,21], and GM plasticity in the ventral PCC [22]. In a meta-analysis study, Melis and colleagues [23] reported that mindfulness training of over 6 weeks alters functional connectivity (FC) between networks involved in attention, executive function, emotional reactivity, and mind wandering. One study found that mindfulness training reduces default mode network (DMN) hyperconnectivity even after just one session [24]. These studies demonstrate plasticity of the brain that occurs in the early stages of mindfulness training and raises the question of its link to the change in one’s mindfulness and mind wandering tendencies.

As for studies involving long-term mindfulness training, long-term meditators were compared to meditation-naïve controls. Across these studies, the ACC has been most consistently linked to mindful training, which is a region that has been found to be largely involved in mind wandering tendencies [25]. Other regions consistently affected by mindfulness training across studies are known to be involved in meta-awareness (frontopolar cortex), body awareness (sensory cortices and insula), memory processes (hippocampus), self and emotion regulation (mid-cingulate cortex and orbitofrontal cortex along with ACC), and intra- and inter-hemispherical communication (superior longitudinal fasciculus and corpus callosum) [25].

Studies have shown that there is a large number of parallels between DMN and mind wandering states, suggesting that they represent similar states of opposition to external perception and having anticorrelation to brain regions that engage in external sensory processes [2]. Meta-analysis of functional neuroimaging studies found that key DMN regions are also involved in mind wandering, including medial PFC, PCC, medial temporal lobe, and bilateral inferior parietal lobule [26]. Notably, a study by Mason and colleagues [3] demonstrated that DMN recruitment is even greater during the periods with a high incidence of mind wandering. It has also been shown that medial PFC and PCC of DMN are relatively deactivated in experienced meditators compared to non-meditators. However, stronger connectivity has been found in mediators both at baseline and during meditation among the dorsolateral PFCs, dorsal ACC, and PCC, which are regions related to self-monitoring and cognitive control [27].

Interestingly, the anterior PFC, the dorsal ACC, and the parahippocampal cortex were found to be more active during mind wandering when participants were unaware of their thoughts rather than aware [28,29]. The evidence showing stronger recruitment of default and executive network regions in mind wandering outside of meta-awareness further suggests that spontaneous and deliberate mind wandering should be considered to be separate variables that engage different brain structures and functions. Previous findings also suggest that mindfulness traits should be treated as possible mediator in studies investigating mind wandering traits, and vice versa, as they share strong causal relations.

Most of the studies mentioned so far investigated mind wandering and mindfulness by comparing groups involved in different variations in mindfulness training that could have affected the subjects in different ways. The frequency of mind wandering in the lab and in daily life also have been found to share a positive correlation, indicating that the mind wandering tendency is a relatively stable individual trait [6,7,30,31,32,33]. Indeed, it has been found that an eight-week mindfulness training induces significant positive change in three of the five individual traits associated with mindfulness defined in the Five Facets Mindfulness Questionnaire (FFMQ) [34]: acting with awareness, nonjudging of inner experience, and observing [30]. Mindfulness traits have also been associated with brain features. The FFMQ facet describing has been associated with the right anterior insula (Brodmann area, or BA 13), the right parahippocampal gyrus/amygdala (BA 28), the right dorsolateral PFC (BA 46), the right inferior parietal lobule (BA 40), and left superior PFC (BA 9) [31,33]; the FFMQ nonjudging of inner experience facet has been positively correlated with the surface area of the right superior PFC (BA 10); the nonreactivity to inner experience facet has been negatively correlated with the thickness of the right superior PFC (BA 8) and middle occipital cortex [33]. In terms of brain function, mindfulness traits as measured by FFMQ were linked to increased FC among neural regions associated with attentional control, interoception, and executive function, and decreased FC among neural regions associated with self-referential processing and mind wandering [35]. In addition, dispositional mindfulness of an individual has been found to be positively correlated with enhanced PFC and attenuated amygdala in emotion-regulation, which suggests that dorsomedial PFC regions are associated with mindfulness skills [36,37].

### 1.2. Current Study

The aim of this study was to model the associations among mindfulness, mind wandering, and morphometric features of the brain through a data fusion machine learning approach. In particular, we investigated whether or not deliberate and spontaneous mind wandering are mediated by one’s mindfulness characteristics. Many studies have so far put in much effort towards better understanding of different types of mind wandering and their neurological attributes. However, there has been no recent study that has directed the investigation towards the mediating effect of mindfulness on mind wandering. The results of our study could also elucidate their possible inverse relationship, which has been a recurring question raised in previous studies. A clear implication from existing evidence is that mind wandering is influenced by DMN connectivity, and that the regions involved in these networks are susceptible to plastic change through mindfulness training that alters one’s mindfulness trait over time. In order to test the effect of mindfulness on mind wandering characteristics beyond existing inferences, we performed a mediation analysis of the trait variables and brain structure covariates composed of GM and WM networks.

In order to quantify and investigate the empirical relationship among mind wandering and mindfulness traits with associated brain regions, we obtained trait scores through respective self-report questionnaires along with structural brain data of our participants. For mind wandering, the scores for deliberate and spontaneous mind wandering were treated as separate variables as they engage different brain regions [28,29].

In recent years, we have witnessed the development and surge of machine learning, a computational intelligence model that builds algorithms to solve a specific task through exploration of patterns and reasoning. In cognitive science, machine learning algorithms are used in analyses such as neural decoding, neural response prediction, and hierarchical modeling [38]. Computational approaches based on machine learning can quantify and decode brain network organization using pattern recognition, as well as perform predictive encoding of brain activities based on supervising metrics. Unlike previous structural studies that examined GM and WM alterations separately, we applied a data fusion approach to include both modalities in one model under the reasonable assumption that both GM and WM play important roles in mind wandering and mindfulness. The combinations of linked data points can capture the patterns of high-dimensional dataset, which univariate approach may fail to detect and miss significant findings [39]. We applied unsupervised machine learning algorithms to the brain imaging data of our participants to decompose the fused GM and WM into naturally grouping covarying networks. We then conducted a mediation analysis to assess the influence of mindfulness on deliberate and spontaneous mind wandering based on this data fusion and network decomposition.

### 1.3. Aim and Hypothesis

The main aim of our study was to find out which GM and WM brain features are associated with mindfulness and mind wandering, and to investigate how mindfulness mediates deliberate and spontaneous mind wandering in terms of these associated brain components. To this end, after decomposing the brain into covarying GM and WM networks, we performed a mediation analysis to see if there are mediating effects of mindfulness on mind wandering traits. Additionally, we investigated if certain mindfulness facets mediate the two forms of mind wandering traits as an extra analysis for completeness. We predicted that specific GM and WM networks consisting of structures that have been consistently linked to mindfulness and mind wandering (such as the insula, the cingulate, the basal ganglia, and frontoparietal attentive regions) would assert influence on mindfulness, which in turn would mediate deliberate and spontaneous mind wandering tendencies. We also predicted that some of the GM and WM networks would exert direct effects on mindfulness, and deliberate and spontaneous mind wandering. In terms of the direction of influence, we expected that both significant GM and WM networks would have positive relationship with mindfulness and deliberate mind wandering, but negative relationship with spontaneous mind wandering.

## 2. Materials and Methods

### 2.1. Participants

The data used in this study was entirely acquired from the MPI-Leipzig Mind-Brain-Body open-access database [40], a project conducted by the Max Planck Institute of Human Cognitive and Brain Sciences in Leipzig, Germany, and approved by the ethics committee of the University of Leipzig (reference number 154/13-ff). The project comprises publicly available behavioral and brain imaging datasets of 318 participants with at least a structural quantitative T1-weighted image and a 15 min resting-state fMRI data (for details of all available data, see [41,42]). All participants underwent medical screening to be eligible for MRI sessions and were also screened for any past and present neuropsychological issues. From the database, we selected 76 participants (33 females, 43 males) for this study based on the inclusion criteria of age (between 20 and 45 years old, *m* = 27.1, *SD* = 5.08), right-handedness, negative drug test screening result, not having any history of neurological or psychiatric diagnosis, and availability of scores for the questionnaires (FFMQ and MW-D/MW-S scales). The entire study was conducted in German. All participants provided written consent. Participant recruitment, screening, MRI acquisition, and the initial release of preprocessed data were performed by the Leipzig group. All subsequent preprocessing, data fusion, and statistical analyses described from Section 2.6 onward were conducted by the current authors.

### 2.2. Deliberate Mind Wandering (MW-D) and Spontaneous Mind Wandering (MW-S) Scales

Deliberate and spontaneous mind wandering scales are self-reported questionnaires that ask the subjects to assess their mind wandering tendencies in everyday life. A German adapted version of MW-D and MW-S scales [43] were used to quantify the subjects’ deliberate and spontaneous mind wandering traits. MW-D and MW-S are separate and distinct traits that involve different degrees of self-control and mechanisms [44]. For instance, MW-D is positively associated with *non-reactivity to inner experience* category of FFMQ, while MW-S is negatively associated with this facet [44]. The original MW-D and MW-S scales are 4-item self-report questionnaires using Likert-scale of 1 to 7 (1: not at all true; 7: very true), and they were tested and validated in previous studies [44,45,46]. The MW-D scale includes items that are related to intentional mind wandering, such as “I allow myself to get absorbed in pleasant fantasy”. The MW-S scale includes items that are related to unintentional mind wandering, such as “I mind-wander even when I’m supposed to be doing something else” [43]. The MW-D and MW-S scales have demonstrated high internal consistency in the original validation studies, with Cronbach’s α ranging from 0.66 to 0.84 for all the questions across samples [43].

### 2.3. Five Facet Mindfulness Questionnaire (FFMQ)

A German translated version of the original English FFMQ was used to measure the subjects’ mindfulness traits. The questionnaire had been developed based on previously existing mindfulness questionnaires and was empirically tested and validated; the original FFMQ validation showed good internal consistency of the subscales with alpha coefficients ranging between 0.75 and 0.9 [34]. The FFMQ is based on conceptualization of mindfulness as a multifaceted construct with five distinct facets represented by five categories: *acting with awareness* (8 items, 40 points), *describing* (8 items, 40 points), *nonjudging of inner experience* (8 items, 40 points), *nonreactivity to inner experience* (7 items, 35 points), and *observing* (8 items, 40 points), with a total of 39 items and 195 points using a Likert-scale of 1 to 5 (1: never or very rarely true; 5: very often or always true). The *acting with awareness* category of the FFMQ (*act_awareness*) measures one’s attentiveness traits with questions such as “When I do things, my mind wanders off and I’m easily distracted.” The *describing* category (*describe*) assesses one’s ability to describe and label one’s sensations, perceptions, thoughts, and feelings with statements such as “I am good at finding words to describe my feelings”. The *nonjudging of inner experience* category (*nonjudge*) measures the one’s tendency to judge one’s own inner experience with questions such as “I criticize myself for having irrational or inappropriate emotions”. The *nonreactivity to inner experience* category (*nonreact*) assesses the level of one’s reactive tendency with questions such as “I perceive my feelings and emotions without having to react to them”. Lastly, the *observing* category of the FFMQ (*observe*) measures one’s self-reported propensity to observe, notice, and attend to sensations, perceptions, thoughts, and feelings by answering statements such as “When I am walking, I deliberately notice the sensations of my body moving”.

### 2.4. Behavioral Data Analysis

The behavioral data was analyzed with JASP (version 17.0; JASP Team, 2023) [47], a platform based on R (R Core Team, 2021) [48] programming language for statistical computing and graphics. To find out if there are significant differences based on demographic characteristics, we compared the participant groups based on gender and age groups for each of the mind wandering (MW-D and MW-S) and mindfulness facet (*act_awareness*, *describe*, *nonjudge*, *nonreact*, and *observe*) scores. Pearson’s correlation coefficients were then calculated to evaluate the relationship among the seven variables.

### 2.5. MRI Data Acquisition/Pre-Processing

The T1-weighted images were acquired on a 3T Siemens Magnetom Verio Scanner equipped with a 32-channel head coil using a MP2RAGE sequence (TR = 5000 ms, TE = 2.92 ms, TI1 = 700 ms, TI2 = 2500 ms, flip angle 1 = 4°, flip angle 2 = 5°, voxel size = 1.0 mm isotropic, duration = 8.22 min). The images were pre-processed with SPM12 (Statistical Parametric Mapping) [49] and CAT12 (Computational Anatomy Toolbox) [50] in the MATLAB (version 9.14; The MathWorks Inc., 2022) [51] environment. A visual check of data quality was performed in order to identify any distortion, such as head motion and other artifacts. The images were oriented to anterior commissure as the origin, and segmented into gray matter (GM), white matter (WM), and cerebrospinal fluid. The processed images were registered with Diffeomorphic Anatomical Registration Through Exponentiated Lie algebra (DARTEL) [52] toolbox for SPM12 and normalized to the MNI (Montreal Neurological Institute) space with a spatial Gaussian smoothing with 12 mm, full-width at half-maximum (FWHM) Gaussian kernel.

### 2.6. Data Fusion and Network Decomposition Using Unsupervised Machine Learning

Pre-processed structural MRI data were decomposed into covarying networks using data-driven parallel independent component analysis (PICA) [53], an unsupervised machine learning approach. PICA is a modified ICA that applies dynamic constraints to both GM and WM modalities simultaneously to assess their interrelationships and decompose the brain into meaningful networks that share the same response pattern. The analysis takes into account both GM and WM structures (by fusing the two modalities) to determine canonical covariate patterns, which allows natural measure of dynamic brain functions and connectivity, and offers improved reliability compared to ICA estimates (with tighter clusters and differently bootstrapped datasets) with lower dimensionality. PICA was conducted with Fusion ICA Toolbox (FIT version 2.0; http://trendscenter.org/software/fit, accessed on 2 April 2023) to fuse GM and WM to find covariate matrix and decompose the brain into independent networks. This multi-model fusion technique identifies brain regions that covary in their properties under the reasonable assumption that they belong to networks involved in carrying out similar psychological functions. To assess the algorithmic reliability of estimated independent components, Icasso [54,55] was run 10 times. Each run generated a set of independent components, which were then clustered across runs. We examined the stability index (Iq) provided by ICASSO, which ranges from 0 (unstable) to 1 (perfect stability). We dropped components with Iq value of less than 0.90 as recommended by literature [54].

### 2.7. Mediation

We conducted mediation analyses to examine the causal relationship among mindfulness and mind wandering (MW-D and MW-S) facets with dependency on the PICA GM and WM networks. We assigned the GM-WM networks as the predictor variable and tested the mind wandering and mindfulness facets as either mediator variable or outcome variable. The analysis was performed with JASP. The mediation models were run as path-analytic SEM. Delta method approximation of standard errors was used to calculate the confidence interval with normal theory of confidence intervals and maximum likelihood estimator.

## 3. Results

### 3.1. Behavioral Data

**Figure 1:**
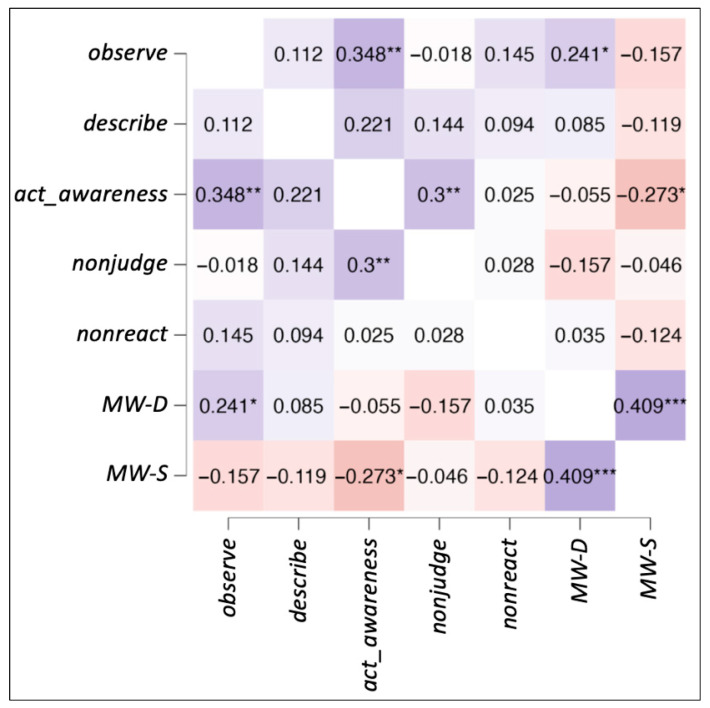
Spearman’s partial correlations heatmap. The figure shows correlations among the five facets of FFMQ, MW-D, and MW-S. Significant values are denoted by * for *p* < 0.06, ** for *p* < 0.01, and *** for *p* < 0.001.

For all the participants, the mean and standard deviation of the FFMQ scores were as follows: *act_awareness* (*M* = 16.7 ± 2.82), *describe* (*M* = 28.8 ± 5.82), *nonjudge* (*M* = 20.8 ± 4.59), *nonreact* (*M* = 18.9 ± 3.54), and *observe* (*M* = 22.2 ± 3.41). For mind wandering scales, the scores were as follows: MW-D (*M* = 3.5 ± 1.05) and MW-S (*M* = 3.2 ± 1.01). Correlations among the behavioral data obtained from the questionnaires were calculated with Spearman’s partial correlation. All correlations among the five variables from the FFMQ, MW-D, and MW-S are shown in Figure 1. Significant positive correlations were found between: *act_awareness* and nonjudge (*r_s_* = 0.300, *p* = 0.009), *act_awareness* and *observe* (*r_s_* = 0.348, *p* = 0.002), *observe* and MW-D (*r_s_* = 0.241, *p* = 0.039), and MW-D and MW-S (*r_s_* = 0.409, *p* < 0.001). Marginally significant positive correlation was also found between *act_awareness* and MW-S (*r_s_* = 0.221, *p* = 0.059). A significant negative correlation was found between *act_awareness* and MW-S (*r_s_* = −0.237, *p* = 0.019).

We also performed *t*-tests and correlations to evaluate the effects of gender and age for the FFMQ facets, MW-D, and MW-S (Table 1).

There was an effect of gender for the mindfulness facet nonjudge (*t* = −2.18, *p* = 0.03). No other significant effect was found.

### 3.2. Data Fusion and Network Decomposition

**Figure 2:**
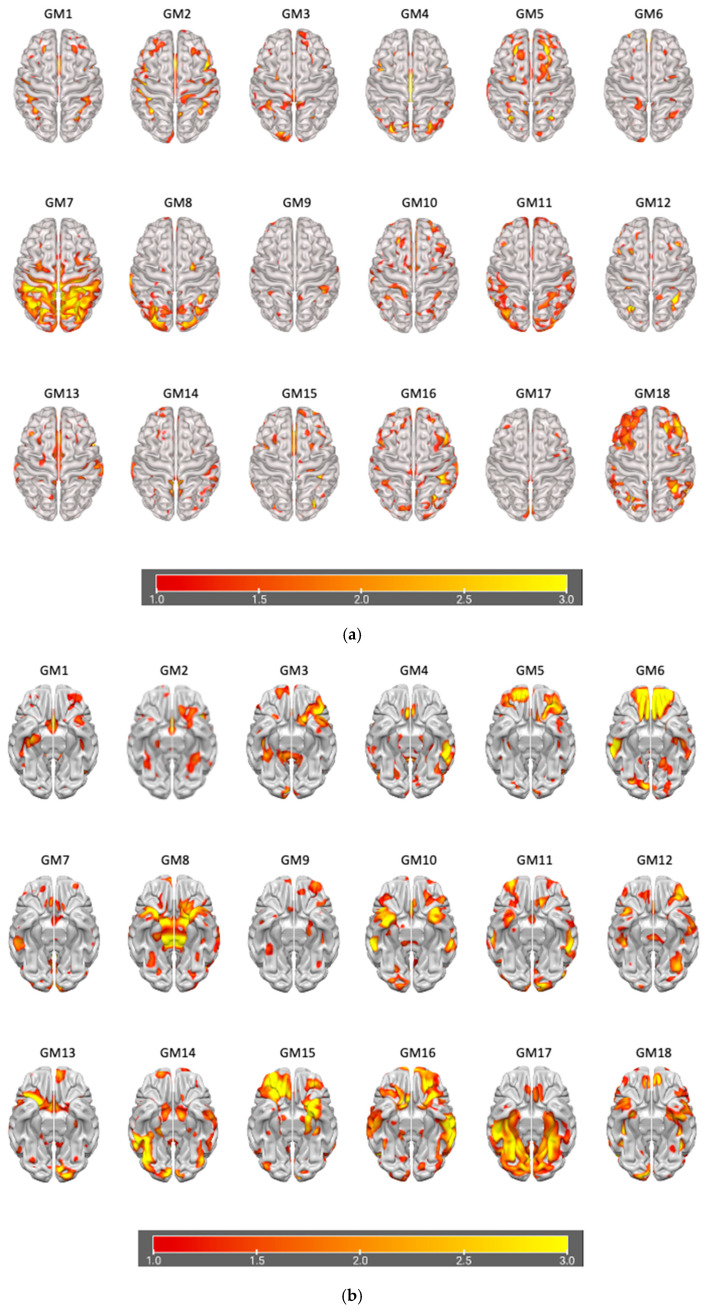
Plots of PICA GM brain networks. (**a**) Superior view (**above**); (**b**) inferior view (**below**). Brain plots of the 18 GM independent covarying networks identified by unsupervised machine algorithms using the information theoretic criteria.

**Figure 3:**
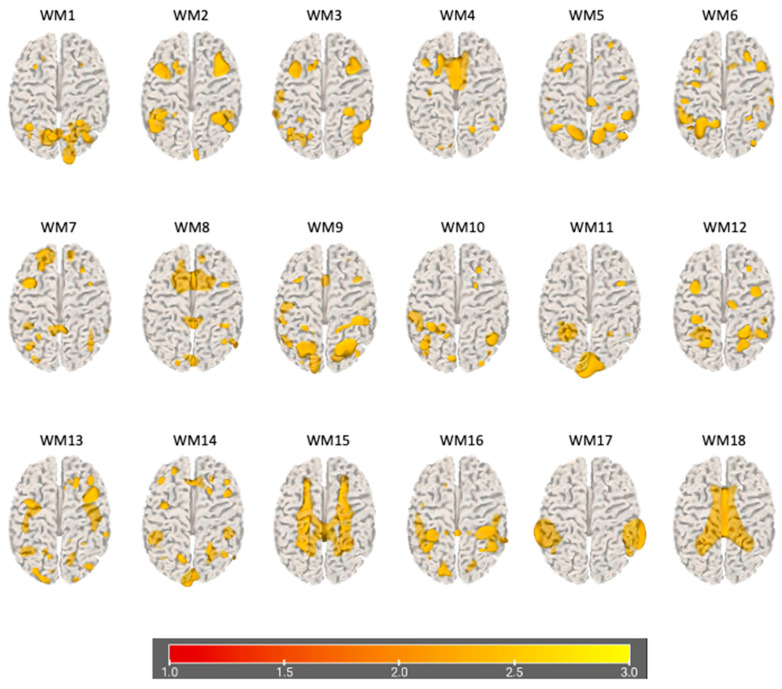
Plots of PICA WM brain networks (3D mesh). The figure shows brain plots of the 18 WM independent covarying networks identified by unsupervised machine algorithms using the information theoretic criteria.

The information theoretic criteria [56] estimated the presence of 18 independent covarying GM and WM networks in our data. The output from PICA consists of a matrix with the number of subjects (rows) and the loading coefficients for each PICA component (columns). The brain plots of all independent component estimates are shown in Figure 2 for the GM networks and Figure 3 for the WM networks.

### 3.3. Mediation Analysis

**Figure 4:**
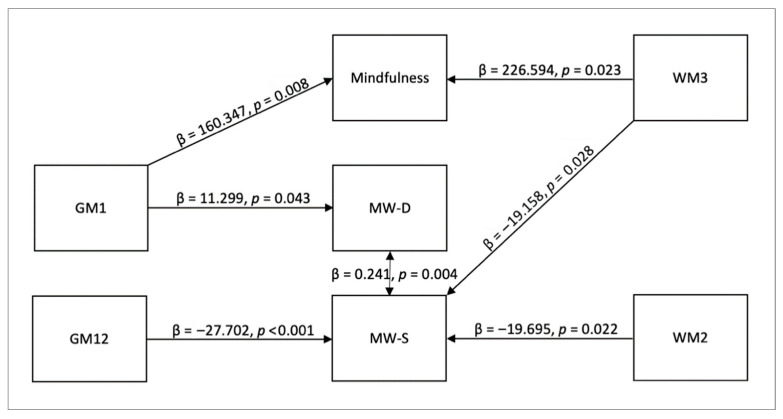
Direct effects among GM and WM networks, mindfulness, and mind wandering. The diagram shows that GM1 has a direct effect on mindfulness and deliberate mind wandering, GM12 and WM2 have a direct effect on spontaneous mind wandering, WM3 has a direct effect on mindfulness and spontaneous mind wandering, and the two forms of mind wandering have a direct effect on each other.

We conducted a mediation analysis to reveal which GM and WM networks are associated with the two forms of mind wandering (MW-D and MW-S), as well as determining if mindfulness plays a mediating role. In this analysis, the effect strength of the mediating variable (i.e., mindfulness measured with FFMQ scores) indicated the strength of its mediating influence among the predictor (i.e., GM and WM networks) and the outcome variables (i.e., MW-D and MW-S). Significant indirect effect indicates that there is an intervening variable that mediates the independent variable’s influence on the dependent variable [57]. The summary of the significant findings reported in the following subsections are also summarized in Figure 4 with direct effects among the GM and WM networks, mindfulness, and mind wandering. Detailed morphometric features of the significant PICA networks are shown in the figures with lists of their positive and negative GM or WM concentrations in the corresponding tables beneath (Table 2, Table 3, Table 4 and Table 5). PICA components with a bilateral volume equal to or below 0.3 cc were removed from the tables in order to focus on more significant components that make up these networks.

Mediation Effect of Mindfulness on MW-D. No indirect effect of mindfulness on MW-D was found. Direct effects indicated that GM1 predicted MW-D (β = 11.299, *SE* = 5.576, *z* = 2.026, *p* = 0.043, 95% CI [0.37, 22.228]). This effect did not survive Bonferroni correction (adjusted threshold *p* < 0.0028), and we therefore report it as exploratory. GM1 also predicted Mindfulness (β = 160.347, *SE* = 60.405, *z* = 2.655, *p* = 0.008, 95% CI [41.955, 278.739]), which also did not survive correction. The residual covariance between MW-D and MW-S was significant (β = 0.241, *SE* = 0.085, *z* = 2.844, *p* = 0.004, 95% CI [0.075, 0.407]), but also exploratory under Bonferroni adjustment.

Mediation Effect of Mindfulness on MW-S. No indirect effect of mindfulness (as measured by the sum of FFMQ scores) on MW-S was found. Direct effect included GM12 → MW-S (β = −27.702, *SE* = 7.29, *z* = −3.8, *p* < 0.001, 95% CI [−41.99, −13.414]), which remained significant after Bonferroni correction. WM2 → MW-S (β = −19.695, *SE* = 8.611, *z* = −2.287, *p* = 0.022, 95% CI [−36.574, −2.817]) and WM3 → MW-S (β = −19.158, *SE* = 8.716, *z* = −2.198, *p* = 0.028, 95% CI [−36.241, −2.075]) were significant at the uncorrected level (*p* < 0.05) but did not survive correction, and are thus reported as exploratory. WM3 also predicted mindfulness (β = 226.594, *SE* = 99.469, *z* = 2.278, *p* = 0.023, 95% CI [31.638, 421.549]), which again did not survive correction. The residual covariance for MW-D ↔ MW-S was significant (β = 0.336, *SE* = 0.105, *z* = 3.2, *p* = 0.001, 95% CI [0.13, 0.542]) after correction.

**Figure 5:**
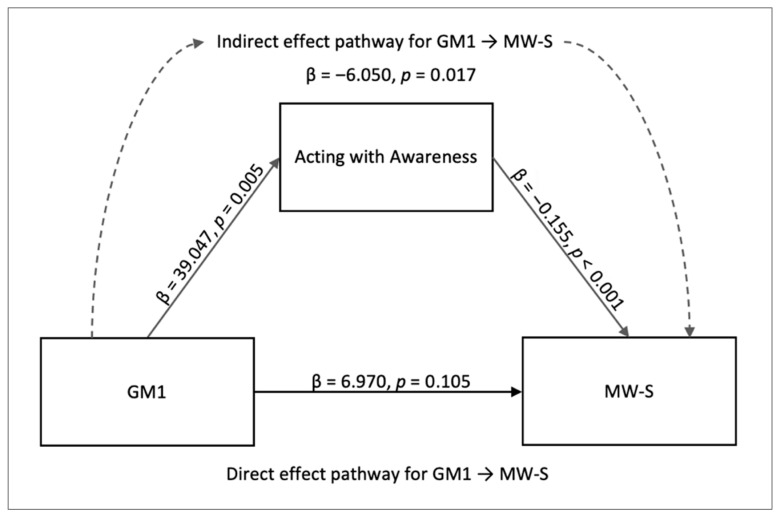
Mediation model for the indirect effect of *acting with awareness* on MW-S. The diagram depicts the mediation effect of *acting with awareness* between GM1 components and spontaneous mind wandering.

**Figure 6:**
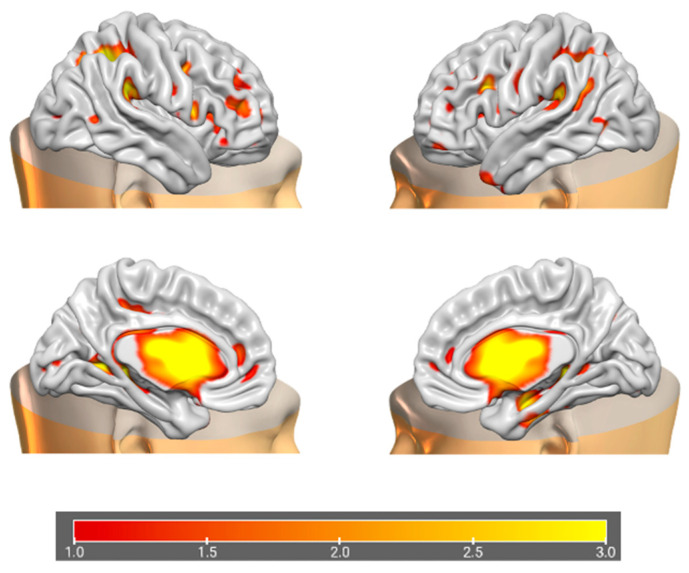
Brain plots depicting morphometric features of GM1 components.

**Figure 7:**
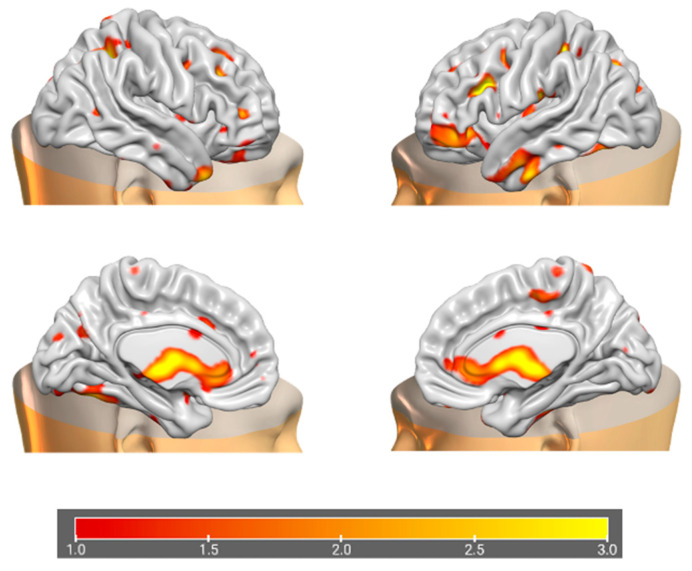
Brain plots depicting morphometric features of GM12 components.

**Figure 8:**
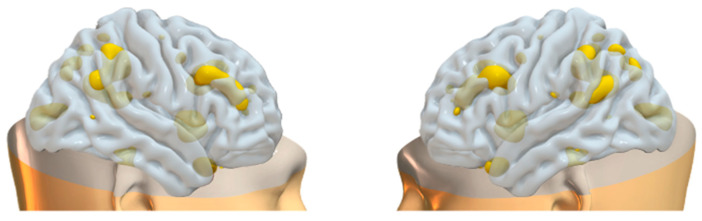
Brain plots depicting morphometric features of WM2 components.

**Figure 9:**
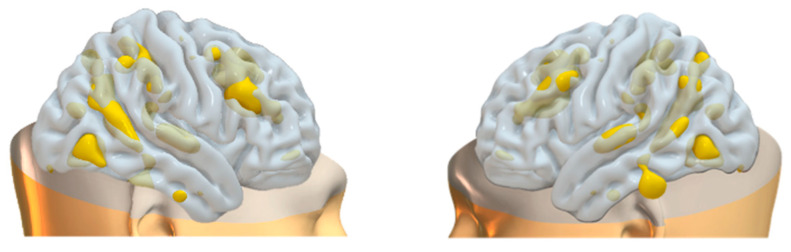
Brain plots depicting morphometric features of WM3 components.

Mediation Effect of Individual Mindfulness Facets on Mind Wandering. For completeness of the analysis, we also ran an additional mediation analysis for each of the mindfulness facets for their influence on mind wandering. In this process, we found a significant indirect mediating effect for the FFMQ facet *acting with awareness*. The indirect effects involved the effect of GM1 on MW-S with *acting with awareness* trait as the moderator (GM1 → *acting with awareness* → MW-S; β = −6.050, *SE* = 2.538, *z* = −2.384, *p* = 0.017, 95% CI [−11.024, −1.076]). This path (Figure 5) was supported by GM1 → *acting with awareness* (β = 39.047, *SE* = 14.034, *z* = 2.782, *p* = 0.005, 95% CI [11.54, 66.553]), and between *acting with awareness* and MW-S (β = −0.155, *SE* = 0.034, *z* = −4.624, *p* < 0.001, 95% CI [−0.221, −0.089]). However, the indirect effect did not survive Bonferroni correction across the five facet-level tests and is reported as exploratory. The direct effect of GM1 → MW-S was not significant (β = 6.970, *SE* = 4.299, *z* = 1.621, *p* = 0.105, 95% CI [−1.456, 15.397]). See Figure 6, Figure 7, Figure 8 and Figure 9 for the relative brain plots.

## 4. Discussion

In the present study, our aim was to find morphometric brain features associated with mindfulness and mind wandering, and to investigate whether mindfulness mediates deliberate and spontaneous mind wandering in terms of these associated brain components. We found effective relationships among mindfulness, deliberate and spontaneous mind wandering, and PICA networks GM1, GM12, WM2, and WM3. In summary, PICA network GM1 was found to have a positive effect on deliberate mind wandering, GM12, WM2, and WM3 were found to have a negative effect on spontaneous mind wandering, and GM1 and WM3 were found to have a positive effect on mindfulness. In addition, WM1 had a positive relationship with the *acting with awareness* component of mindfulness, which was found to mediate spontaneous mind wandering. This mediation aligns with previous findings demonstrating a negative relationship between *acting with awareness* and spontaneous mind wandering [58].

Our results further indicated a positive relationship between deliberate and spontaneous mind wandering. As a side note, GM1 correlated with WM5, and GM12 correlated with WM8 in the PICA fusion results; however, these WM counterparts were not significantly associated with the effects of interest in the mediation analysis. One possible explanation for this counterintuitive finding is that the correlations between GM1 and WM5, as well as GM12 and WM8, were negative. In the PICA fusion results, WM2 and WM3 were not correlated with any GM component.

A key finding of this study is the role of mindfulness—specifically the *acting with awareness* facet—in mediating the relationship between GM1 and spontaneous mind wandering. GM1 predominantly involves increased gray matter in subcortical structures, which supports previous research suggesting a top-down or bottom-up mindful emotion regulation mechanisms within limbic areas [14,15,16]. These findings also align with prior studies demonstrating gray matter changes in the hippocampus [30,31,59,60,61,62], PCC [30,61], temporo-parietal junction [30], and cerebellum [30,63] following mindfulness training. Previous studies also found that the caudate volume is negatively associated with dispositional mindfulness measured through the MAAS (Mindful Attention Awareness Scale) [32,64] and it was shown to be reduced through a 5-week mindfulness training in risky drivers [65]. It has also been found that there is a negative relationship in terms of FC between the thalamus and PCC (measured through the MAAS scores), suggesting that these brain areas are involved in switching between mind wandering and mindfulness [66].

The GM1 network also includes reduced gray matter volume in the cingulate. However, we found a positive, rather than a negative relationship between the GM1 network and mindfulness scores. This could be explained by the fact that the mindfulness factor in our study is based on the FFMQ rather than the MAAS scores. Our results are also consistent with the findings of a meta-analysis study that show increased volume and activation of the thalamus, as well as increased brain activation of the caudate and in long-term meditators [67]. Results from other studies also support that mindfulness training results in increased volume of the caudate [68,69]. The caudate is suggested to be involved in the alteration of habitual direction of attention [69,70,71,72], while the thalamus is suggested to be involved in the orientation of attention and conscious awareness [73]. These findings are coherent with our results showing positive relationship between GM1 and mindfulness. Furthermore, our mediation analysis shows that this network mainly involving the caudate and the thalamus is associated with *acting with awareness*, which can alter the process of mind wandering by influencing attentional processes. The GM12 network components also included brain structures previously discussed to be implicated in mindfulness and mind wandering, including the cerebellum, dorsolateral PFC, inferior parietal lobule, precuneus, and fusiform gyrus. The components are involved in mindfulness and mind wandering often through closely related functions such as self-referential processing, attentional processes, and other higher cognitive functions as well as interoceptive roles. Known modulatory role of the brain components in our significant networks well illustrate how these networks would directly or indirectly influence mindfulness and mind wandering facets in our study.

The maladaptive form of mind wandering can manifest as a cumbersome symptom in psychiatric diseases, and also exacerbated by factors such as aging, hyperactivity, time of the day, and depression [74]. Some studies even found negative correlation between mind wandering and happiness [5,45,75,76,77]. Seneca expressed this notion in Letter to Lucilius (Letter 78): “To be happy, two elements must be rooted out—the fear of future suffering, and the recollection of past suffering”. In this vein, studies suggest that the frequency of mind wandering in daily life might have an inverse relationship with mindfulness [6]. Aforementioned studies have shown that mindfulness is positively linked to awareness (e.g., towards interoceptive and exteroceptive cues) and emotion regulation skills. We also mentioned that mindfulness training can improve a person’s mindfulness abilities after only a short session. Our study demonstrates that these changes can be represented by morphometric features of the brain to support behavioral reports, which can be useful in a clinical setting to measure short- and long-term results of a psychological intervention. As an example of practical application, a recent study showed that reduced activation of left cuneus is linked to positive outcome of cognitive behavior therapy across many studies, along with decreased activity in medial PFC and ACC [78]. Our findings could also be of interest in the studies of large-scale brain networks and mindfulness. The GM and WM networks identified in this study overlap with systems implicated in the regulation of internally directed thought, particularly in terms of DMN and its interaction with frontoparietal control and salience networks. Specifically, GM1 (caudate and thalamus) and the frontoparietal WM networks may influence structural substrates that support control over DMN driven activity. This interpretation is consistent with recent work by Sorella et al. [58], which demonstrated that temporal variability of the DMN selectively predicts spontaneous mind wandering, which in turn is negatively associated with the mindfulness facet *acting with awareness*. Together, these findings suggest that mindfulness may reduce spontaneous mind wandering by strengthening frontoparietal and subcortical control mechanisms that modulate DMN activity. Our structural findings complement functional network evidence by showing that the mediation through *acting with awareness* can be understood within a broader large-scale network framework of mindfulness and mind wandering.

## 5. Conclusions

Our study provides novel insights into the morphometric brain features associated with mindfulness, deliberate mind wandering, and spontaneous mind wandering, as well as the mediating effects of the FFMQ facet *acting with awareness* on spontaneous mind wandering. The covarying GM and WM networks in this study consisted of many brain structures that were linked to mindfulness and mind wandering in previous studies, but it is still unclear how and which of these structural components exert influence on one’s mindfulness and mind wandering traits. The FFMQ and MW-D/MW-S instruments used in the study have been validated extensively, with demonstrated internal consistency in prior work [34,43]. Nonetheless, future work could benefit from replication with longer or more robustly validated scales. Although the sample size was modest for complex brain network analysis, effect sizes are reflected in the standardized coefficients and confidence intervals we report. Sensitivity analysis in G*Power 3.1.9.7 to quantify the minimal effect size detectable with our design and sample indicated that our study is sensitive to small-to-medium effects. Replication in larger datasets would strengthen stability assessment. The confidence intervals were computed with the delta method implemented in JASP. Although bootstrap CIs are often recommended with small samples, we retained the delta method for consistency and note this as a limitation. Our study also showed for the first time how mindfulness affects spontaneous mind wandering in a general population sample, complementing whole-brain data-driven predictive modeling approaches that focus on group-level effects [79]. Future studies should aim to investigate individual-level structural and functional brain changes to establish biomarkers for monitoring the effectiveness of psychological interventions. Additionally, longitudinal studies with experienced meditators and clinical populations would further elucidate the mediating effects of mindfulness on spontaneous mind wandering.

## Figures and Tables

**Table 1:** Effects of age and gender. Mean and SD of the scores for females and males; independent samples *t*-test for gender differences; Spearman’s correlations for age differences; * *p* < 0.05.

	Female		Male		Gender		Age	
	(*n* = 33)		(*n* = 43)		(*df* = 74)		(*n* = 76)	
	*M*	*SD*	*M*	*SD*	*t*	*p*	*rho*	*p*
Mindfulness								
act_awareness	16.15	2.600	17.16	2.94	−1.56	0.12	−0.22	0.05
describe	30.12	5.441	27.86	5.97	1.70	0.09	−0.05	0.69
nonjudge	19.52	4.078	21.77	4.76	−2.18	0.03 *	0.05	0.68
nonreact	18.42	3.446	19.23	3.60	−0.99	0.33	−0.06	0.59
observe	22.94	3.142	21.58	3.52	1.75	0.09	0.04	0.71
Mind Wandering								
MW-D	3.42	1.146	3.54	0.98	−0.45	0.653	0.05	0.69
MW-S	3.30	1.045	3.07	0.99	1.00	0.322	0.09	0.42

**Table 2:** GM1 network brain components. The first column indicates the name of the brain area, the second column indicates the name according to the Brodmann classification, the third column indicates the volume of gray or white matter concentration, and the fourth column indicates the coordinates of the peak for the area.

Area	Brodmann Area	Volume (cc)	Max Value, MNI Left/Right (x, y, z)
Caudate	32	3.8/2.8	7.9 (−9, 12, 10)/6.4 (10, 8, 15)
Thalamus	23, 30	1.5/1.5	6.9 (−10, −21, 16)/6.1 (13, −24, 15)
Caudate	24, 32	4.4/3.7	6.7 (−3, 10, 5)/6.0 (12, 4, 18)
Thalamus	30, 31	2.4/2.0	6.2 (−15, −28, 14)/5.6 (21, −35, 9)
Parahippocampal Gyrus	30	0.3/0.1	5.6 (−24, −38, 5)/4.8 (24, −39, 3)
Hippocampus/Caudate	30, 31	0.4/0.6	4.7 (−27, −38, 2)/4.4 (30, −36, 1)
Precuneus	7	0.0/0.4	0 (0, 0, 0)/4.3 (18, −60, 43)

**Table 3:** GM12 network brain components. The first column indicates the name of the brain area, the second column indicates the name according to the Brodmann classification, the third column indicates the volume of gray or white matter concentration, and the fourth column indicates the coordinates of the peak for the area.

Area	Brodmann Area	Volume (cc)	Max Value, MNI Left/Right (x, y, z)
Cerebellar Tonsil	37	0.5/3.3	4.9 (−37, −38, −38)/9.2 (49, −50, −40)
Broca/Visual Motor	7, 45, 46	1.5/1.0	6.2 (−37, 20, 21)/8.0 (31, −52, 39)
Inferior Parietal Lobule	7	0.0/0.7	0 (0, 0, 0)/6.2 (36, −53, 39)
Precuneus	7	0.8/0.1	6.1 (−28, −62, 36)/4.2 (25, −50, 44)
Tuber	19, 37	0.4/1.0	4.1 (−46, −74, −25)/5.9 (55, −47, −29)
Fusiform Gyrus	18, 19	0.0/0.4	0 (0, 0, 0)/5.3 (27, −87, −18)
Inferior Semi-Lunar Lobule	37	0.0/0.4	0 (0, 0, 0)/5.3 (53, −60, −35)
Culmen	37	0.0/0.4	0 (0, 0, 0)/5.0 (50, −44, −29)
Declive	19	0.1/0.6	3.8 (−46, −74, −22)/4.8 (31, −85, −18)

**Table 4:** WM2 network brain components. The first column indicates the name of the brain area, the second column indicates the name according to the Brodmann classification, the third column indicates the volume of gray or white matter concentration, and the fourth column indicates the coordinates of the peak for the area. The software (FIT) computes WM nomenclature according to the adjacent GM components. Therefore, the areas reported for WM components should be referred to only as reference for the corresponding coordinates.

Area	Brodmann Area	Volume (cc)	Max Value, MNI Left/Right (x, y, z)
Middle Frontal Gyrus	9, 46	1.7/2.0	9.1 (−37, 17, 28)/8.6 (37, 18, 31)
Superior Temporal Gyrus	39	1.1/0.1	8.6 (−42, −51, 25)/3.6 (48, −55, 29)
Supramarginal Gyrus	39, 40	1.5/0.8	8.0 (−45, −51, 27)/5.0 (48, −52, 32)
Supramarginal Gyrus	7, 39	1.7/2.6	7.4 (−39, −48, 25)/7.8 (34, −40, 39)
Inferior Parietal Lobule	7, 39	0.9/1.2	7.8 (−45, −48, 25)/7.4 (37, −42, 42)
Culmen	20, 37	0.8/0.2	7.1 (−46, −39, −28)/4.4 (45, −41, −27)
Cerebellar Tonsil	20, 37	0.6/0.8	6.9 (−43, −40, −35)/6.4 (43, −40, −37)
Precentral Gyrus	9, 46	0.1/0.3	4.1 (−43, 19, 35)/6.3 (37, 21, 34)

**Table 5:** WM3 network brain components. The first column indicates the name of the brain area, the second column indicates the name according to the Brodmann classification, the third column indicates the volume of gray or white matter concentration, and the fourth column indicates the coordinates of the peak for the area. The software (FIT) computes WM nomenclature according to the adjacent GM components. Therefore, the areas reported for WM components should be referred to only as reference for the corresponding coordinates.

Area	Brodmann Area	Volume (cc)	Max Value, MNI Left/Right (x, y, z)
Middle Frontal Gyrus	9, 46	1.9/1.5	9.5 (−37, 19, 32)/5.7 (40, 25, 25)
Precentral Gyrus	9	0.6/0.1	8.1 (−37, 16, 35)/4.0 (36, 19, 34)
Visual Association/Insula	9, 19, 46	1.2/2.6	5.7 (−27, −70, −2)/6.3 (37, 22, 25)
Inferior Temporal Gyrus	19	0.2/0.4	4.8 (−42, −67, 2)/6.3 (43, −67, 1)
Superior Temporal Gyrus	37, 44	0.7/1.3	4.5 (−56, −13, 1)/6.3 (50, −50, 16)
Middle Occipital Gyrus	19	0.2/0.4	4.6 (−37, −65, 3)/5.8 (40, −65, 3)
Inferior Parietal Lobule	7	0.1/0.3	4.0 (−30, −42, 55)/5.1 (36, −34, 42)
Middle Temporal Gyrus	37, 39	0.1/0.3	3.5 (−55, −53, 11)/4.6 (43, −61, 27)

## Data Availability

The data used in this study was entirely acquired from the MPI-Leipzig Mind-Brain-Body open-access database: https://openneuro.org/datasets/ds000221/versions/1.0.0 accessed on 15 February 2023 [40].

## Abbreviations

The following abbreviations are used in this manuscript:

ACC	anterior cingulate cortex
BA	Brodmann area
DMN	default mode network
FC	functional connectivity
FFMQ	five facet mindfulness questionnaire
GM	gray matter
ICA	independent component analysis
MAAS	Mindful Attention Awareness Scale
MRI	magnetic resonance imaging
PCC	posterior cingulate cortex
PFC	prefrontal cortex
MW-D	deliberate mind wandering
MW-S	spontaneous mind wandering
PICA	parallel independent component analysis
WM	white matter
